# Tumor microenvironment-driven immune evasion in triple-negative breast cancer: mechanisms, clinical challenges, and therapeutic opportunities

**DOI:** 10.3389/fimmu.2026.1897496

**Published:** 2026-09-28

**Authors:** Hui Zhang, Kun Zhou, Na Li, Hui Duan, Jie Hou, Danxin Wu

**Affiliations:** 1 Department of Endocrinology and Rheumatology, Taihe Hospital, Hubei University of Medicine, Shiyan, Hubei, China; 2 Department of Breast Thyroid Vascular Surgery, Taihe Hospital, Hubei University of Medicine, Shiyan, Hubei, China; 3 Department of Hematology, Taihe Hospital, Hubei University of Medicine, Shiyan, Hubei, China; 4 Department of Emergency Medicine, Taihe Hospital, Hubei University of Medicine, Shiyan, Hubei, China; 5 Department of Oncology, Taihe Hospital, Hubei University of Medicine, Shiyan, Hubei, China; 6 Key Laboratory of Cancer Therapy Resistance and Clinical Translational Study, Shiyan, Hubei, China

**Keywords:** breast cancer immunotherapy, immune checkpoint inhibitors, immune evasion, immune suppression, PD-L1, therapeutic resistance, triple-negative breast cancer, tumor microenvironment

## Abstract

Triple-negative breast cancer (TNBC) is an aggressive and clinically challenging breast cancer subtype defined by the absence of estrogen receptor, progesterone receptor, and HER2 expression. Because these classical therapeutic targets are lacking, chemotherapy remains a central systemic treatment; however, TNBC is frequently associated with early recurrence, distant metastasis, immune escape, and therapeutic resistance. Growing evidence shows that these malignant features are strongly shaped by the tumor microenvironment (TME), a complex network of tumor cells, extracellular matrix, stromal cells, immune cells, vascular structures, cytokines, chemokines, and signaling mediators. Compared with hormone receptor-positive breast cancer, TNBC often displays stronger immune infiltration and higher immune-checkpoint activity, suggesting an immune-inflamed phenotype in many cases. Nevertheless, immune-cell presence does not always produce effective antitumor immunity because TNBC can develop PD-L1 upregulation, alternative checkpoint activation, antigen-presentation loss, T-cell exhaustion, macrophage polarization, regulatory T-cell expansion, myeloid-derived suppressor cell recruitment, stromal immune exclusion, hypoxia, metabolic stress, epigenetic regulation, and exosome-mediated immune suppression. This review summarizes TME-driven immune evasion in TNBC, emphasizing molecular heterogeneity, hot and cold immune phenotypes, immunosuppressive cellular networks, PD-1/PD-L1 signaling, stromal barriers, metastatic immune escape, advances in clinical immunotherapy, adverse effects, and emerging TME-directed strategies. Understanding these mechanisms may support rational combination therapies, biomarker-guided patient selection, and individualized immunotherapeutic approaches for TNBC.

## Graphical Abstract

**Figure:**
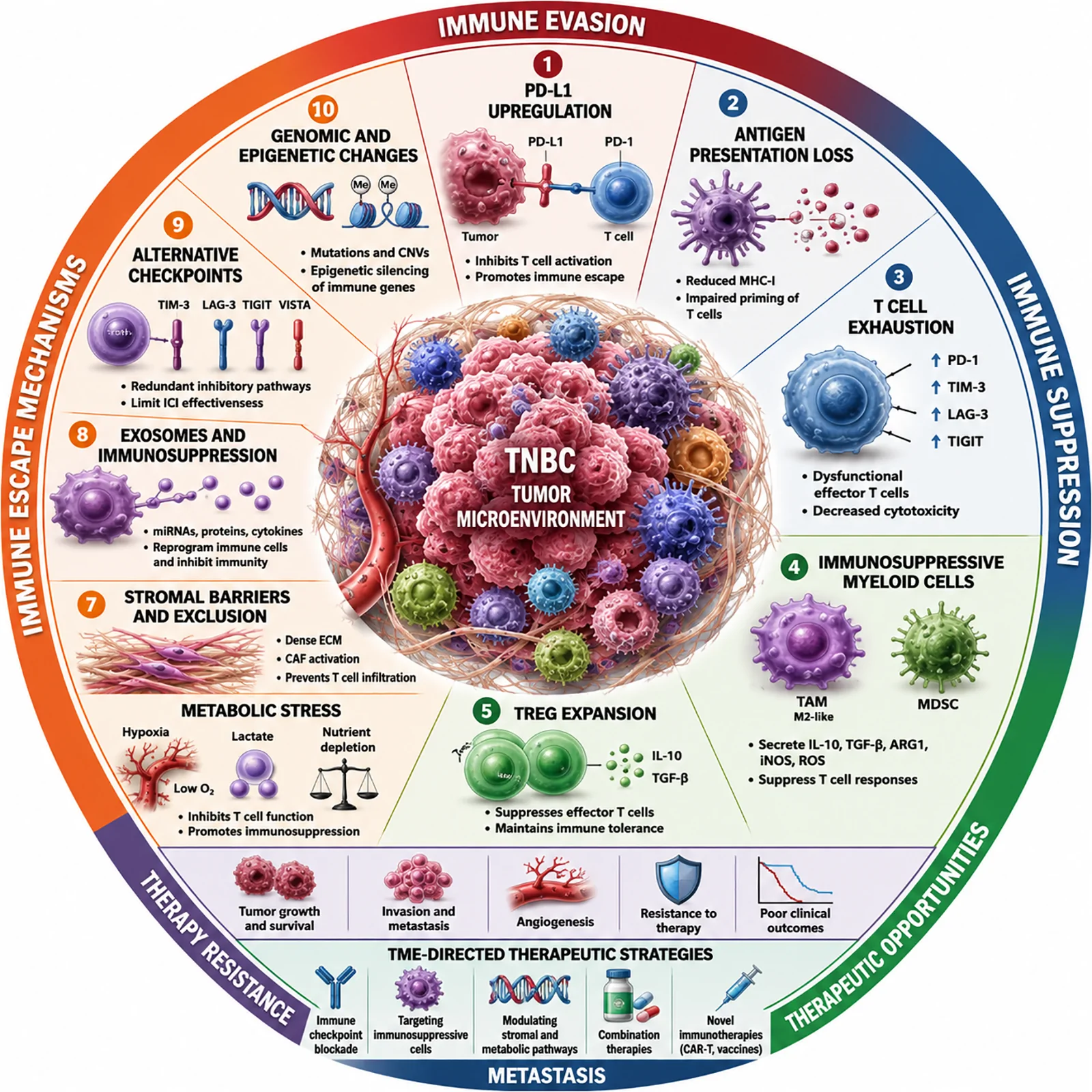
Overview of tumor microenvironment–driven immune evasion in triple-negative breast cancer (TNBC), including immune checkpoints, antigen presentation loss, T-cell exhaustion, stromal and metabolic suppression, and exosome-mediated signaling, leading to immune escape, therapy resistance, and metastasis.

## 1 Introduction

Breast cancer is the most frequently diagnosed malignancy among women worldwide. It remains a major contributor to cancer-related mortality, creating a substantial global health burden despite major progress in screening, early diagnosis, and treatment (1–3). Over the past two decades, breast cancer management has shifted from a largely anatomy-based treatment model toward a biomarker-driven approach involving molecular classification, surgery, radiotherapy, chemotherapy, endocrine therapy, HER2-targeted therapy, antibody–drug conjugates, immunotherapy, and increasingly individualized precision oncology strategies (4–7). Breast cancer is therefore understood as a biologically heterogeneous group of diseases rather than a single pathological entity. Molecular and genomic studies have identified distinct intrinsic subtypes with different gene-expression patterns, metastatic behavior, therapeutic vulnerabilities, and clinical outcomes (8–13). Among these subtypes, triple-negative breast cancer (TNBC) is widely recognized as one of the most aggressive and difficult to treat because it lacks estrogen receptor (ER), progesterone receptor (PR), and human epidermal growth factor receptor 2 (HER2) expression, thereby excluding the routine use of endocrine therapy and HER2-directed therapy (14–17). Although chemotherapy remains an important systemic treatment and may produce an initial clinical response, TNBC is frequently associated with high proliferative activity, early recurrence, visceral metastasis, distant relapse, drug resistance, and poorer survival compared with many other breast cancer subtypes (18–20).

TNBC accounts for approximately 10%–20% of breast cancer cases and displays marked molecular diversity, genomic instability, immune heterogeneity, and clinically aggressive behavior. These unfavorable characteristics are not determined by tumor-cell intrinsic alterations alone; rather, they are strongly shaped by the surrounding tumor microenvironment, which functions as a dynamic ecosystem composed of malignant cells, stromal cells, extracellular matrix components, endothelial cells, fibroblasts, adipocytes, immune cells, cytokines, chemokines, metabolites, extracellular vesicles, and multiple signaling mediators (21–25). Tumor cells and the TME interact bidirectionally: tumor cells remodel the local microenvironment to support survival and immune escape, while the TME provides biochemical, mechanical, metabolic, vascular, and immunological signals that promote tumor proliferation, invasion, angiogenesis, metastasis, therapeutic resistance, and immunosuppression (26). Therefore, understanding the biological and immunological organization of the TNBC microenvironment is essential for explaining why this subtype behaves aggressively and why current therapeutic approaches remain insufficient for many patients.

The importance of the TME is particularly evident in the context of breast cancer immunotherapy. Immunotherapy has become a major therapeutic strategy in oncology. It has shown clinically meaningful activity in selected patients with TNBC, particularly in tumors with immune-inflamed features or when combined with chemotherapy (27–29). However, the efficacy of immunotherapy depends strongly on tumor subtype, disease stage, antigenicity, tumor-infiltrating lymphocyte abundance, PD-L1 expression, antigen-presentation capacity, T-cell priming, myeloid-cell composition, stromal organization, and the broader immunosuppressive state of the TME (30). TNBC is generally more immunogenic than hormone receptor-positive breast cancer because it often displays higher tumor-infiltrating lymphocyte levels, increased immune-related gene expression, greater PD-L1 expression, and higher tumor mutational burden (31). Nevertheless, many TNBC tumors remain poorly responsive to, or resistant to, immunotherapy because immune infiltration may coexist with T-cell exhaustion, regulatory T-cell recruitment, suppressive myeloid-cell activity, macrophage-mediated tumor promotion, PD-L1 upregulation, extracellular matrix barriers, metabolic restriction, and adaptive immune checkpoint activation.

In the context of breast cancer immune evasion, TNBC provides an important model for understanding how tumors can remain clinically aggressive despite visible immune infiltration. Unlike hormone receptor-positive breast cancer, which is often relatively immune-cold, TNBC frequently exhibits an immune-inflamed phenotype with stronger interferon-related signaling, higher cytotoxic lymphocyte infiltration, and increased immune checkpoint activity. However, this immune activity is often counterbalanced by suppressive myeloid cells, regulatory T cells, exhausted cytotoxic T cells, cancer-associated fibroblasts, extracellular matrix remodeling, hypoxia, nutrient competition, metabolic stress, and checkpoint-mediated inhibition (32). Therefore, TNBC illustrates a central challenge in breast cancer immunology: the presence of immune cells does not necessarily indicate functional anti-tumor immunity. This review is positioned within the broader question of how breast cancer cells and their microenvironment evade immune surveillance and how these mechanisms can be therapeutically reversed through rational combination strategies and biomarker-guided immunotherapy. Although immune evasion is relevant across breast cancer subtypes, this review deliberately focuses on TNBC because TNBC combines high clinical aggressiveness with pronounced immune heterogeneity, frequent checkpoint activation, variable immune infiltration, and clinically meaningful but inconsistent responses to immunotherapy. This subtype, therefore, provides a useful disease model for analyzing how tumor-intrinsic evolution, suppressive immune-cell networks, stromal remodeling, metabolic stress, and therapeutic pressure converge to produce immune escape. By using TNBC as a focused framework, the review addresses the broader question in breast cancer immunology of why immune recognition fails and how immune resistance may be reversed through rational, biomarker-guided, TME-directed strategies.

## 2 Molecular heterogeneity of TNBC and its relationship with immune response

TNBC is a highly heterogeneous breast cancer subtype with diverse gene-expression profiles, molecular features, pathological characteristics, and clinical behaviors. It is associated with high invasiveness, rapid recurrence, early metastasis, and poor prognosis (33). TNBC is more common in younger women and shows relatively higher prevalence among African American and Hispanic populations (34). At present, chemotherapy remains the central systemic therapeutic option for TNBC, but treatment responses differ substantially among patients. This variability is closely linked to the molecular diversity of TNBC, including genomic instability, chromosomal aberrations, differential pathway activation, immune heterogeneity, and distinct stromal features (35). Therefore, TNBC should not be viewed as a uniform disease. Instead, molecular classification provides an important foundation for understanding disease progression, predicting therapeutic response, and identifying patients who may benefit from chemotherapy, immunotherapy, targeted therapy, or combination strategies. This heterogeneity is also reflected in the immune phenotype of TNBC. Some TNBC tumors are relatively “hot,” showing abundant T-cell infiltration, interferon pathway activation, immune-checkpoint expression, and greater potential sensitivity to immune checkpoint blockade. Other tumors are “cold” or immune-excluded, with weak antigen presentation, low immune-cell recruitment, poor cytotoxic T-cell penetration, dense stromal barriers, suppressive myeloid-cell infiltration, and limited response to immunotherapy. Between these two extremes, many TNBC tumors display an intermediate immune state in which immune cells are present but functionally suppressed, as shown in **Figure 1**. This distinction is important because the clinical success of immunotherapy depends not only on whether immune cells are present, but also on whether these cells can recognize tumor antigens, enter tumor nests, avoid exhaustion, and maintain cytotoxic activity within the TME (36–38).

**Figure 1:**
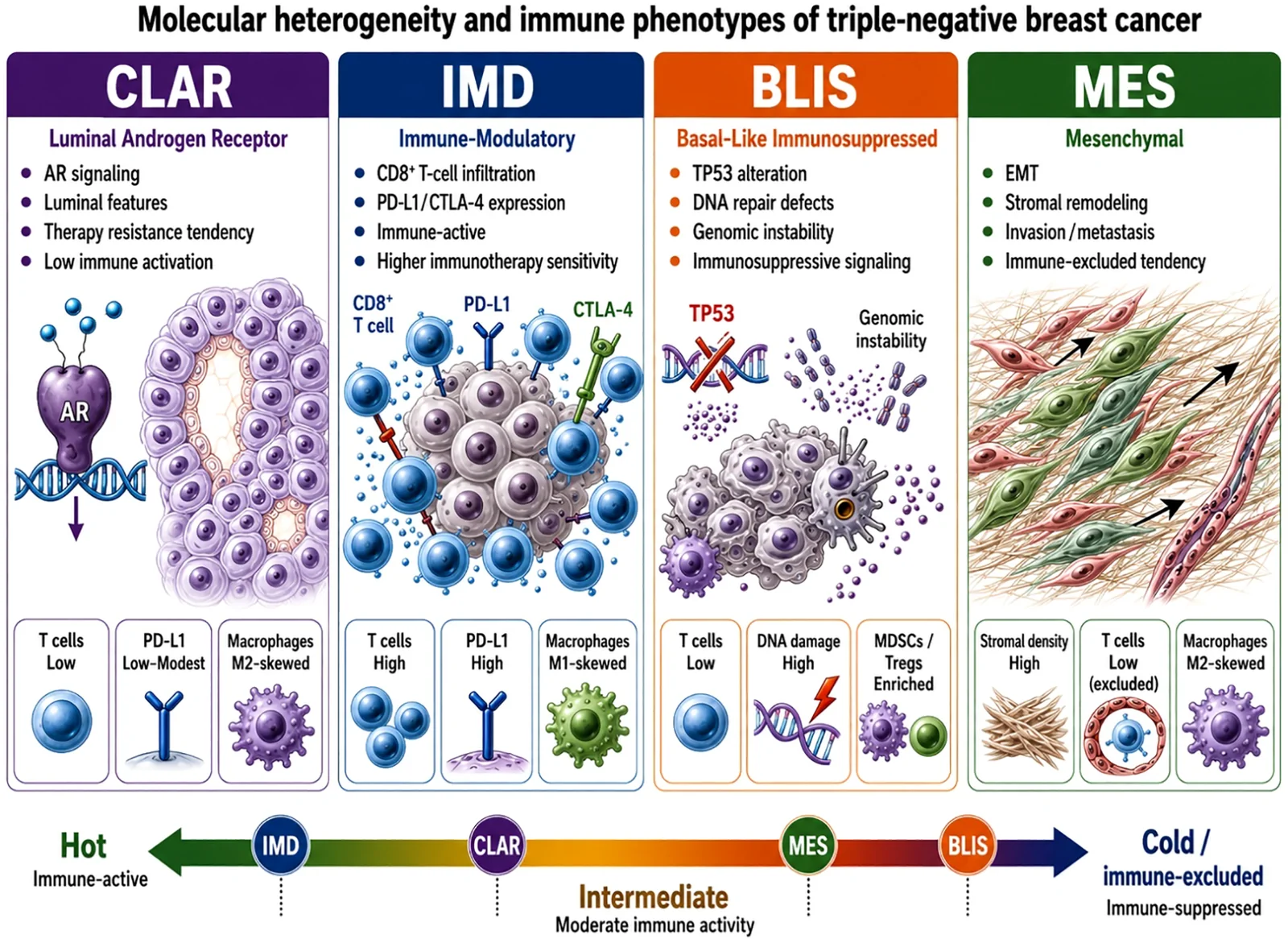
Molecular heterogeneity and immune phenotypes of triple-negative breast cancer. TNBC molecular subtypes (LAR, IM, BLIS, MES) and their associated immune phenotypes, immune-evasion mechanisms, and immunotherapy sensitivity.

To better illustrate the heterogeneity of TNBC and its clinical implications for immune evasion and immunotherapy response, the major molecular subtypes are summarized in **Table 1**.

**Table 1:** Molecular subtypes of TNBC, immune phenotypes, immune escape mechanisms, and immunotherapy sensitivity.

TNBC subtype	Immune phenotype	Major immune escape mechanisms	Immunotherapy sensitivity
LAR (Luminal Androgen Receptor)	Immune-cold	Low TIL infiltration, androgen receptor signaling, reduced antigen presentation	Low
IM (Immunomodulatory)	Immune-hot	PD-1/PD-L1 adaptive resistance, CTLA-4 signaling, high T-cell infiltration	High
BLIS (Basal-like Immunosuppressed)	Immune-excluded	TP53 mutation, MHC-I downregulation, T-cell dysfunction	Low–Moderate
MES (Mesenchymal)	Immune-excluded	CAF activation, EMT signaling, TGF-β–mediated stromal barrier	Low
BL1/BL2	Mixed	DNA repair defects, genomic instability, PD-L1 upregulation	Moderate–High

TNBC molecular subtypes demonstrate distinct immune landscapes that strongly influence tumor–immune interactions and therapeutic response. Immune-hot subtypes, particularly immunomodulatory tumors, exhibit high tumor-infiltrating lymphocyte density and enhanced sensitivity to immune checkpoint blockade. In contrast, mesenchymal and basal-like immunosuppressed subtypes are dominated by stromal activation, epithelial–mesenchymal transition, and defective antigen presentation, leading to immune exclusion and resistance to immunotherapy. Luminal androgen receptor tumors are characterized by low immune activation and reduced antigen presentation capacity, resulting in poor responsiveness to immune checkpoint inhibitors. Collectively, these subtype-specific immune profiles highlight the necessity of precision-based immunotherapeutic strategies in TNBC rather than uniform treatment approaches. Based on large-scale genomic and transcriptomic analyses, TNBC has been classified into four major molecular subtypes according to distinct gene characteristics: luminal androgen receptor type (LAR), immune-modulatory type (IM), basal-like immunosuppressed type (BLIS), and mesenchymal type (MES) (39). These subtypes differ significantly in their biological features, immune landscapes, signaling pathways, and potential treatment vulnerabilities. The androgen receptor-related subtype is characterized by androgen receptor expression and represents a clinically important subgroup of TNBC. In patients, androgen receptor-positive TNBC accounts for approximately 10%–53% of cases (40). Androgen receptor expression is often detected in smaller tumors or tumors without lymph-node metastasis, but it may also be associated with chemotherapy insensitivity and drug resistance. In advanced disease, high androgen receptor expression may indicate poor prognosis (41). These findings suggest that androgen receptor signaling may contribute to TNBC progression in a context-dependent manner and may provide a possible therapeutic target for selected patients.

The immune regulation subtype has a distinct immune microenvironment and is characterized by increased CD8+ T-cell infiltration and relatively high expression of immune checkpoint molecules, including CTLA-4 and PD-L1 (42, 43). This subtype may be more sensitive to immune checkpoint inhibitors because the presence of cytotoxic immune infiltration and immune checkpoint activation suggests a pre-existing anti-tumor immune response that may be reactivated by immunotherapy. In contrast, the basal-like immunosuppressive subtype is the most genomically unstable subtype, with a high mutation rate in the tumor suppressor gene TP53 and frequent deletions in genes involved in DNA repair (44). This subtype also shows downregulation of immune regulatory pathways and cytokine pathways involving T cells, B cells, and natural killer cells. These features suggest that the basal-like immunosuppressive subtype may escape immune surveillance through impaired immune activation and weakened anti-tumor immune signaling. The mesenchymal subtype is relatively genetically stable and shows high expression of breast cancer-related signaling pathways. This subtype may be associated with stromal remodeling, epithelial–mesenchymal transition, cellular plasticity, invasion, and metastatic potential. Patients who achieve pathological complete remission after neoadjuvant chemotherapy usually have a favorable prognosis (45). Therefore, molecular subtyping of TNBC is valuable not only for understanding tumor biology but also for predicting which patients may benefit from neoadjuvant therapy, immune checkpoint blockade, or other individualized therapeutic approaches.

Recent evidence further shows that TNBC heterogeneity should be interpreted not only through fixed molecular subtype classification but also through the dynamic processes of clonal evolution and immune selection (46). Intratumoral heterogeneity allows different tumor-cell clones to express distinct neoantigens, immune-checkpoint molecules, metabolic programs, and antigen-presentation capacities (47). Under immune or therapeutic pressure, clones with immune-evasive phenotypes may be preferentially selected, leading to a tumor population that is less visible to cytotoxic T cells and more resistant to immune checkpoint blockade. This process can occur through antigen-dependent mechanisms, such as loss of shared neoantigens, downregulation of MHC-I, B2M alteration, and impairment of antigen-processing machinery, as well as antigen-independent mechanisms, including PD-L1 upregulation, recruitment of regulatory T cells, myeloid-derived suppressor cells, and M2-like macrophages, stromal exclusion of T cells, and metabolic competition with effector immune cells (48). Therefore, TNBC heterogeneity is not only a diagnostic or classification issue but also a major biological driver of immune evasion and immunotherapy resistance (49). The concept of immunoediting provides an additional framework for understanding how TNBC evolves toward immune escape (50). During the elimination phase, innate and adaptive immune responses can recognize and destroy immunogenic tumor cells. During the equilibrium phase, residual tumor cells may persist under immune pressure while acquiring less immunogenic phenotypes. During the escape phase, tumor cells actively evade immune recognition by reducing antigen presentation, expressing inhibitory immune checkpoint molecules, recruiting suppressive immune populations, and remodeling the TME into an immune-resistant niche (51). More recent interpretations describe TNBC immune evasion through a “three C” model: camouflage, evasion of immune recognition; coercion, active suppression of immune effector function; and cytoprotection, resistance to immune-mediated cytotoxicity. This model is useful because it integrates tumor-cell intrinsic mechanisms with metabolic, epigenetic, and microenvironmental changes that collectively determine whether TNBC behaves as an immune-responsive or immune-resistant tumor.

## 3 Tumor microenvironment remodeling and TNBC progression

The TME of TNBC is a highly organized yet abnormal biological system comprising tumor cells, extracellular matrix components, stromal cells, cancer stem cells, immune cells, cancer-associated fibroblasts, vascular networks, and multiple soluble signaling molecules (52). These components interact continuously, creating a microenvironment that supports tumor survival, growth, invasion, angiogenesis, immune escape, and metastatic dissemination. In malignant tumors, the extracellular matrix is frequently remodeled and disorganized. This abnormal extracellular matrix can provide signals that promote tumor initiation, tumor-related angiogenesis, inflammation, and the development of a tumor-promoting microenvironment (53). In TNBC, extracellular matrix remodeling may also influence immune cell migration, drug penetration, tissue stiffness, and tumor cell motility. By creating both biochemical and physical barriers, the extracellular matrix can limit the effective infiltration of immune cells and contribute to therapeutic resistance (**Figure 2**).

**Figure 2:**
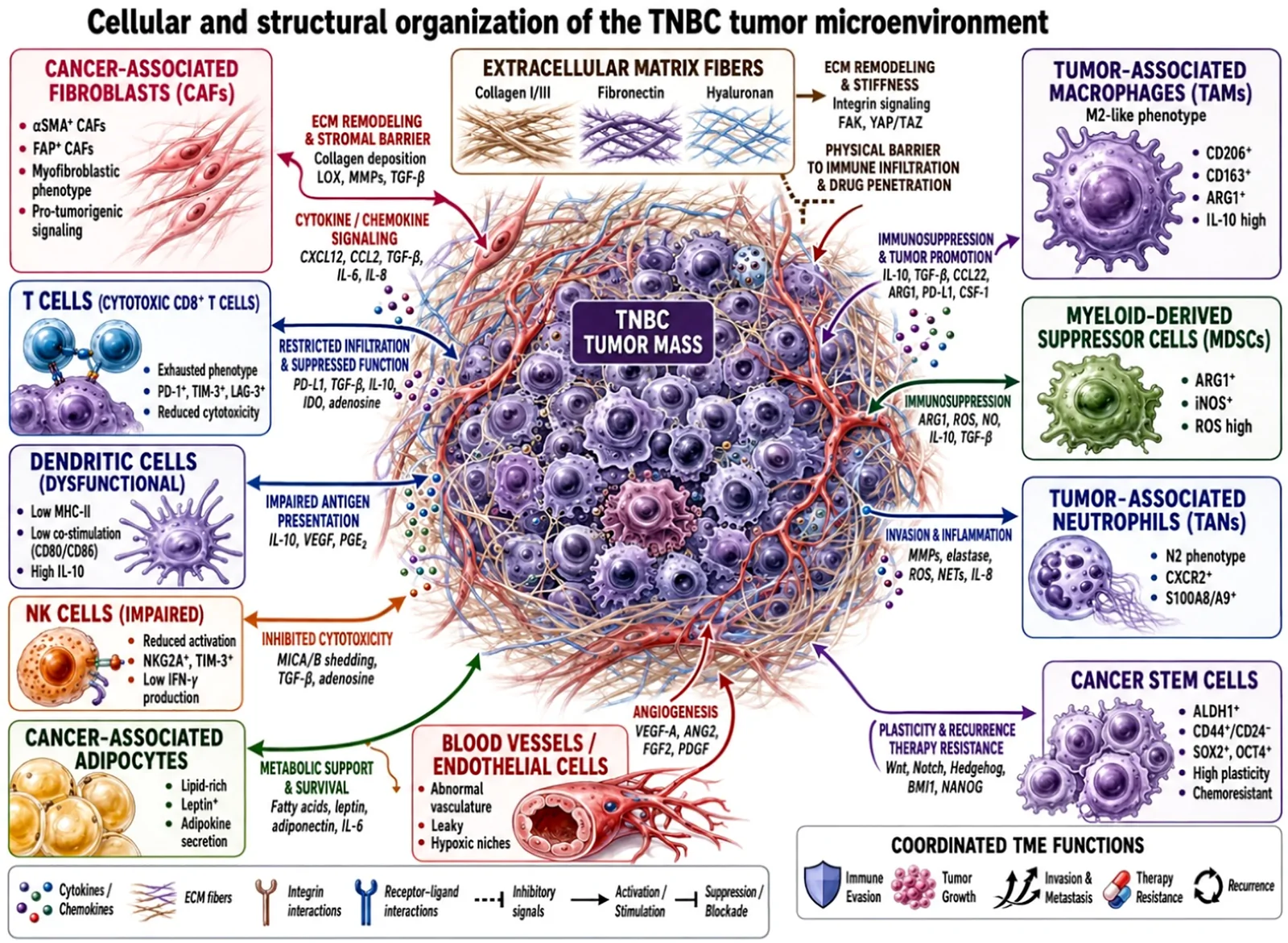
Cellular and structural organization of the tumor microenvironment in triple-negative breast cancer. Illustration of the complex TNBC tumor microenvironment composed of tumor cells, cancer-associated fibroblasts (CAFs), immune cells (T cells, macrophages, dendritic cells, NK cells), cancer stem cells, endothelial cells, adipocytes, and extracellular matrix (ECM) components. The image highlights dynamic interactions among these compartments that regulate tumor progression, immune evasion, angiogenesis, metabolic adaptation, and metastasis. Special emphasis is placed on ECM remodeling, stromal activation, and physical immune exclusion barriers that limit cytotoxic T-cell infiltration and contribute to therapeutic resistance.

Immune evasion in triple-negative breast cancer (TNBC) is a highly dynamic and stepwise evolutionary process that progresses in parallel with tumor development from early preneoplastic lesions to invasive and metastatic disease. In the earliest stages, immune surveillance is primarily mediated by innate immune cells and emerging adaptive T-cell responses that can eliminate transformed or pre-malignant cells. However, during early tumor evolution, selective immune pressure drives the emergence of tumor cell clones with reduced immunogenicity through mechanisms such as defects in antigen presentation, adaptation of early interferon signaling, and initial immune checkpoint activation (54, 55). As lesions progress toward invasive carcinoma, the tumor microenvironment becomes increasingly immunosuppressive, characterized by the expansion of regulatory T cells, the accumulation of myeloid-derived suppressor cells, the polarization of tumor-associated macrophages toward an M2-like phenotype, and the activation of cancer-associated fibroblasts that promote extracellular matrix remodeling and T-cell exclusion (56, 57). At the same time, tumor cells acquire enhanced metabolic fitness and begin to exploit hypoxia-driven pathways, including HIF-1α signaling, to sustain survival and immune resistance (23). In advanced and metastatic TNBC, immune escape becomes fully established and is reinforced by persistent PD-L1 expression, widespread T-cell exhaustion, stromal barrier formation, metabolic competition, and exosome-mediated immunosuppressive signaling (58). Collectively, this evolutionary trajectory reflects a transition from immune containment to immune escape, ultimately resulting in a highly suppressive tumor microenvironment that supports tumor progression, therapeutic resistance, and metastatic dissemination.

Cancer-associated fibroblasts are among the most important stromal cells in the TME and play an indispensable role in TNBC progression. Cancer-associated fibroblasts contribute to immune evasion in TNBC through extensive extracellular matrix (ECM) remodeling, metabolic reprogramming, and rewiring of cytokine networks. By altering ECM composition and increasing tissue stiffness, they create a physical barrier that restricts cytotoxic T-cell infiltration into tumor nests. In addition, CAFs actively reshape tumor metabolism by promoting glycolytic flux and nutrient competition within the tumor microenvironment, thereby limiting immune cell function (59). Through secretion of cytokines and chemokines such as TGF-β, IL-6, CXCL12, and CCL2, CAFs reprogram immune cell recruitment and polarization, favoring immunosuppressive phenotypes including regulatory T cells and M2-like macrophages. Collectively, these mechanisms position CAFs as central orchestrators of immune exclusion and therapeutic resistance in TNBC. They can release cytokines, chemokines, growth factors, and extracellular matrix components through autocrine and paracrine mechanisms, thereby strongly influencing tumor behavior and reshaping the surrounding microenvironment (60). Through these secreted factors, cancer-associated fibroblasts promote tumor-cell proliferation, invasion, metastasis, angiogenesis, and survival. They may also contribute to immune evasion by producing immunosuppressive cytokines, altering extracellular matrix structure, limiting infiltration by cytotoxic immune cells, and promoting the recruitment of immunosuppressive populations. In this way, cancer-associated fibroblasts act as active regulators of both tumor progression and anti-tumor immune suppression. Cancer stem cells also contribute to the aggressive behavior of TNBC. These cells are present in many solid tumors and possess strong self-renewal and differentiation capacities. Unlike normal stem cells, cancer stem cells are not adequately regulated by physiological control mechanisms and can continuously generate heterogeneous tumor cell populations (61, 62). They are considered an important source of malignant tumor cells and tumor heterogeneity. Cancer stem cells also contribute to chemotherapy resistance, recurrence, and distant metastasis (63). In the TNBC microenvironment, cancer stem cells may interact with immune cells, fibroblasts, macrophages, and extracellular matrix components to maintain a protective niche that supports immune escape and therapeutic resistance. Their ability to survive under stress conditions, resist cytotoxic therapy, and regenerate tumor populations after treatment makes them an important target for future therapeutic strategies.

The immune microenvironment of TNBC is particularly distinctive, often containing abundant tumor-infiltrating lymphocytes and macrophages. B cells and T lymphocytes are central components of anti-tumor immune defense. CD4+ helper T cells regulate immune responses and assist B-cell activation, while CD8+ cytotoxic T cells recognize and kill target cells. In many tumors, higher levels of tumor-infiltrating lymphocytes are associated with stronger anti-tumor immunity and may predict better treatment response. However, immune-cell infiltration alone does not guarantee tumor elimination. The function, phenotype, localization, and exhaustion status of immune cells are equally important. In TNBC, infiltrating immune cells may be functionally suppressed by immune checkpoint signaling, regulatory T cells, tumor-associated macrophages, inhibitory cytokines, metabolic stress, hypoxia, or extracellular matrix barriers (64). As a result, the tumor may appear immunologically active but remain resistant to effective immune clearance.

Tumor-associated macrophages are another key immune component of the TNBC microenvironment. Macrophages can display different phenotypes and functions depending on local microenvironmental signals. Some macrophage subsets may participate in tumor-cell killing via inflammatory mediators, whereas M2-like macrophages can promote tumor growth, angiogenesis, invasion, metastasis, and immunosuppression by secreting chemokines or growth factors (65). Tumor-associated macrophages and tumor-infiltrating lymphocytes are therefore considered important prognostic factors in cancer. Their density, phenotype, and spatial distribution can strongly affect disease progression and treatment outcomes. In TNBC, the balance between anti-tumor immune activation and immunosuppressive macrophage activity may help determine whether a tumor remains sensitive to immunotherapy or develops immune-evasive behavior. In addition to tumor-associated macrophages and lymphocytes, other immune and stromal populations contribute to the suppressive TNBC microenvironment. Myeloid-derived suppressor cells inhibit T-cell and natural killer cell function through arginine depletion, reactive oxygen species, nitric oxide, suppressive cytokines, and metabolic competition. Regulatory T cells restrict cytotoxic T-cell activation and maintain immune tolerance within the tumor bed. Dysfunctional dendritic cells weaken antigen presentation and reduce effective T-cell priming, while impaired natural killer cells lose their capacity to eliminate tumor cells with reduced MHC expression. Tumor-associated neutrophils may further support angiogenesis, invasion, and immune suppression, particularly when polarized toward pro-tumor phenotypes. Cancer-associated adipocytes can also supply metabolic substrates and inflammatory mediators that promote tumor progression and immune dysfunction. Together, these cellular interactions demonstrate that TNBC immune evasion is maintained by a coordinated network of tumor, stromal, and immune-cell populations rather than by a single suppressive pathway (66–70).

## 4 TME-mediated immune evasion in TNBC

The immune system continuously performs immune surveillance by recognizing and eliminating abnormal or malignant cells. However, tumor cells can acquire multiple strategies to avoid immune recognition and destruction. This process of immune evasion is a central feature of cancer progression. It is particularly important in TNBC because immune activity and immune suppression often coexist within the same tumor microenvironment. The components of the TME contribute to immune escape through several mechanisms, including recruitment of immunosuppressive cells, induction of immune checkpoint molecules, suppression of cytotoxic T-cell activity, alteration of antigen presentation, secretion of inhibitory cytokines, metabolic competition, and creation of physical barriers to immune-cell infiltration (71). Similar immunosuppressive microenvironmental remodeling has also been observed in other solid tumors, including ovarian cancer, highlighting conserved mechanisms of immune escape across malignancies (72). Regulatory T cells, tumor-associated macrophages, myeloid-derived suppressor cells, cancer-associated fibroblasts, exhausted T cells, and negative regulatory molecules such as PD-1 and PD-L1 all contribute to the development of an immunosuppressive niche that allows TNBC cells to survive immune attack. In addition to PD-1/PD-L1 signaling, TNBC immune escape involves a broader immune-checkpoint network. CTLA-4/B7 signaling suppresses early T-cell activation by competing with CD28-mediated costimulation. At the same time, emerging checkpoints such as LAG-3, TIM-3, TIGIT, and CD47-SIRPα contribute to T-cell exhaustion, impaired NK-cell function, macrophage phagocytosis resistance, and adaptive resistance to PD-1/PD-L1 blockade. CD47 is particularly important because it functions as a “don’t eat me” signal that protects tumor cells from macrophage-mediated phagocytosis. These alternative checkpoints help explain why some TNBC tumors remain resistant even when PD-L1 is expressed or when PD-1/PD-L1 inhibitors are used (**Figure 3**). Therefore, immune evasion in TNBC should be interpreted as a coordinated inhibitory network rather than a single-checkpoint process (73, 74).

**Figure 3:**
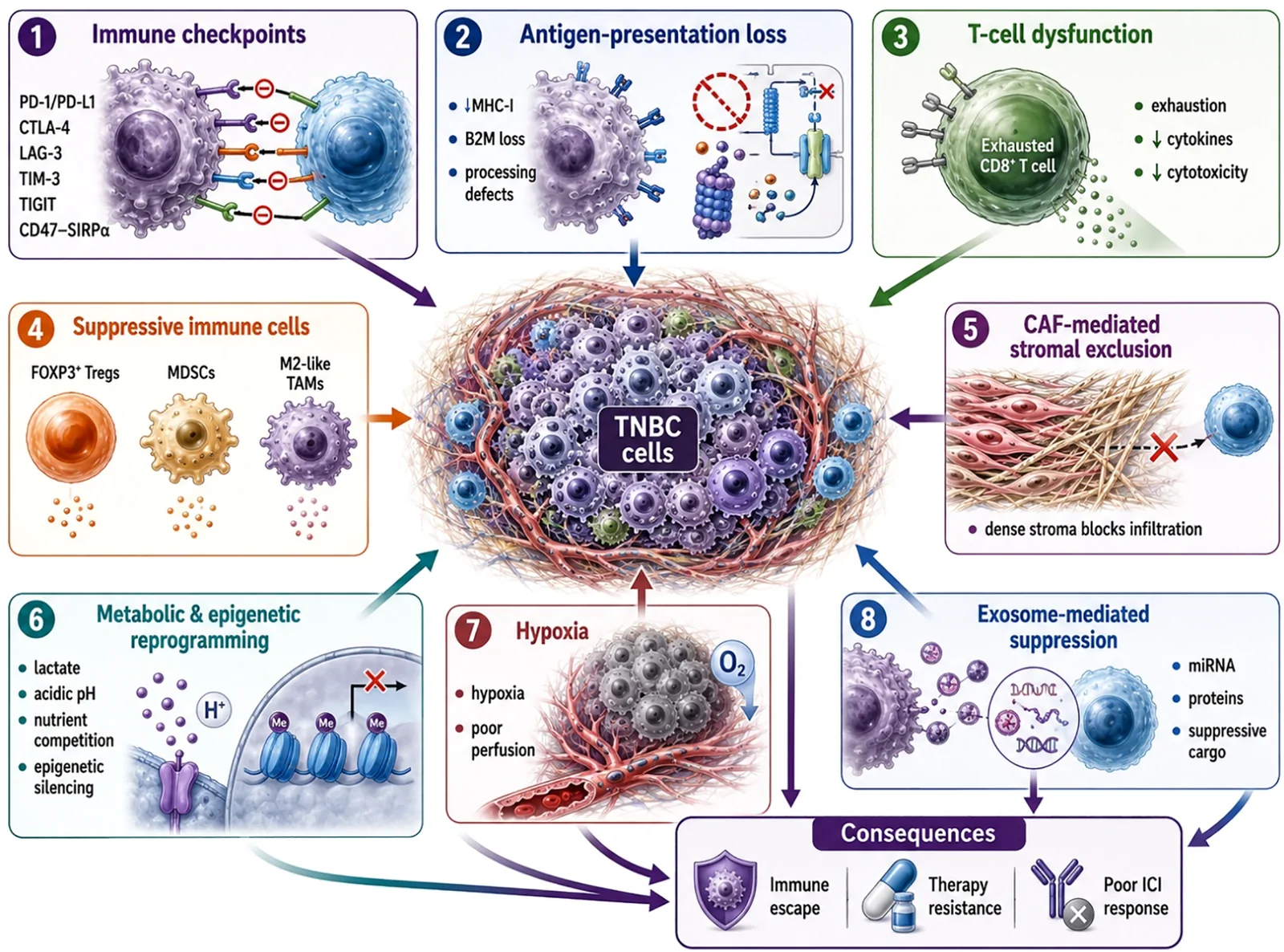
Mechanisms of tumor microenvironment–mediated immune evasion in triple-negative breast cancer. From (1-8) Overview of major immune-evasion mechanisms in TNBC, including immune checkpoint signaling (PD-1/PD-L1, CTLA-4, LAG-3, TIM-3, TIGIT, CD47–SIRPα axis), antigen presentation loss (MHC-I downregulation and β2M alterations), T-cell exhaustion, suppressive immune-cell recruitment (Tregs, MDSCs, M2-like macrophages), CAF-mediated stromal exclusion, metabolic and epigenetic reprogramming, hypoxia-driven signaling, and exosome-mediated immunosuppression. The image integrates these pathways into a coordinated network that drives immune escape, therapeutic resistance, and tumor progression.

### 4.1 CTLA-4 and emerging immune checkpoints

The immune microenvironmental phenotype of TNBC can be divided into three heterogeneous clusters with distinct immune-evasion mechanisms. Cluster 1 is characterized by deficient recruitment of innate immune cells; cluster 2 is associated with insufficient chemotaxis of innate immune cells and low tumor antigen load; and cluster 3 is characterized by high expression of immune checkpoint molecules (75). This classification highlights an important point: TNBC does not evade immunity through a single universal mechanism. Different tumors may evade immune attack through different biological routes, including poor immune-cell recruitment, low antigenicity, defective innate immune activation, or excessive checkpoint-mediated suppression. This heterogeneity partly explains why immune checkpoint inhibitors benefit only a subset of TNBC patients and why combination strategies are needed to convert immune-cold or immune-suppressed tumors into more responsive tumors.

### 4.2 PD-1/PD-L1 immune checkpoint axis

One of the most important mechanisms of immune evasion in TNBC is the upregulation of programmed cell death ligand 1. PD-L1 is an immunosuppressive protein commonly expressed on tumor and immune cells in the TME and can mediate immune evasion by interacting with PD-1 on T cells (76). Through this interaction, PD-L1 suppresses T-cell activation, reduces cytotoxic function, and allows tumor cells to avoid immune clearance. Tumor cells can use PD-L1 upregulation as an adaptive response to immune pressure, and PD-L1 has been considered a poor prognostic indicator in several tumor types, including breast cancer (77). The regulation of PD-L1 expression in TNBC is complex and involves inflammatory cytokines, oncogenic signaling, epigenetic regulation, metabolic stress, and exosome-mediated communication between tumor and immune cells. In the TNBC microenvironment, PD-L1 expression can be induced by several cytokines, among which interferon-γ is one of the strongest inducers (78). This reflects the dual role of interferon-γ in cancer immunity. On one side, interferon-γ can inhibit tumor formation by regulating immune responses and the cell cycle. On the other side, sustained interferon-γ signaling can promote adaptive immune resistance by increasing PD-L1 expression and enabling tumor cells to escape immune surveillance. Several molecular regulators contribute to this process. Histone deacetylase 2 is significantly upregulated in TNBC compared with other breast cancer subtypes and can promote PD-L1 expression in TNBC cells by activating the JAK-STAT1 pathway (79). The E3 ubiquitin ligase UBR5 also plays a crucial role in interferon-γ-induced PD-L1 transcription in TNBC (80). MUC1-C, an oncogenic protein aberrantly expressed in TNBC cells, can also contribute to escape from tumor immune surveillance through the interferon-γ pathway (81). These findings indicate that PD-L1 expression is not only an immune checkpoint event but also a consequence of broader oncogenic, inflammatory, and epigenetic signaling networks (82).

Metabolic abnormalities in the TME further strengthen immune evasion. Hypoxia, low pH, and nutrient deprivation are common features of malignant tumors and can induce endoplasmic reticulum stress (83).

### 4.3 Non-coding RNAs (miRNAs, lncRNAs) in immune regulation

Under endoplasmic reticulum stress, breast cancer cells can release exosomes containing miR-27a-3p. Exosomal miR-27a-3p can upregulate PD-L1 expression in macrophages and promote immune evasion of breast cancer cells by activating the PTEN-AKT/PI3K pathway (84). Emerging evidence also suggests that exosome-mediated immune regulation can extend beyond miRNA transfer, contributing to broader immunomodulatory reprogramming of the tumor microenvironment (85). This mechanism shows how tumor cells communicate with macrophages and other immune cells to create an immunosuppressive microenvironment. It also indicates that exosomal microRNAs may act as important mediators of immune escape and may provide new therapeutic targets for reversing immunotherapy resistance.

### 4.4 DNA methylation and histone modification in immune escape

Metabolic and epigenetic reprogramming further strengthens immune evasion in TNBC. Enhanced glycolysis increases lactate accumulation and extracellular acidification, which can suppress cytotoxic T cells, impair natural killer cell activity, reduce dendritic-cell function, and support regulatory immune populations. Hypoxia-inducible factor 1-alpha (HIF-1α) is a central regulator linking metabolic reprogramming with immune evasion in TNBC. Regulation of ferroptosis and PI3K/AKT/mTOR signaling has also been implicated in TNBC progression, linking metabolic stress responses to tumor survival and immune escape (86). Under hypoxic conditions, stabilization of HIF-1α promotes a shift toward aerobic glycolysis, enhancing glucose uptake and lactate production, which acidifies the tumor microenvironment and suppresses cytotoxic T-cell and natural killer cell activity. This metabolic adaptation strengthens tumor survival while simultaneously impairing anti-tumor immunity. In addition, HIF-1α regulates genes involved in angiogenesis and immune checkpoint expression, further reinforcing immune escape. Metabolomic alterations in amino acid metabolism, including depletion of tryptophan, arginine, and glutamine, restrict T-cell activation and proliferation, while lipid metabolic reprogramming supports immunosuppressive macrophage polarization and tumor progression. Recent studies have also shown that MAEL-mediated metabolic reprogramming can enhance breast cancer progression through mitochondrial enzyme degradation and altered energy metabolism (87). Competition for amino acids such as tryptophan, arginine, and glutamine can also weaken immune-cell proliferation and effector function, while lipid metabolic rewiring supports tumor growth, macrophage polarization, and immune suppression. Epigenetic mechanisms add another layer of immune escape by altering the expression of genes involved in antigen processing, MHC class expression, cytokine signaling, immune checkpoint regulation, and macrophage-mediated phagocytosis. DNA methylation, histone modification, and non-coding RNAs may silence immune-activating pathways while promoting suppressive signaling within the TME. These mechanisms show that immune evasion in TNBC is not only cellular and structural but also deeply connected to metabolic adaptation and epigenetic plasticity (88–92).

### 4.5 T-cell dysfunction and immune exhaustion

T cells in the TME are central to cancer immune surveillance and anti-tumor immunity. CD8+ cytotoxic T cells can directly kill tumor cells, while CD4+ helper T cells coordinate immune responses and support other immune-cell populations. In TNBC, tumor-infiltrating lymphocytes are often considered favorable immune indicators and may be associated with better response to some therapies. However, T-cell infiltration does not always result in effective tumor control because T cells may become exhausted, excluded, or functionally suppressed within the TME. Exhausted T cells often express inhibitory receptors and show reduced cytokine production, proliferation, and cytotoxic activity. The TNBC microenvironment can further impair T-cell function through hypoxia, acidic pH, nutrient deprivation, suppressive cytokines, regulatory T cells, tumor-associated macrophages, and immune checkpoint pathways. Therefore, restoring T-cell activity and reversing T-cell exhaustion remain major goals of immunotherapy in TNBC (93).

Overall, TME-mediated immune evasion in TNBC is a multifactorial and interconnected process. PD-L1 upregulation, interferon-γ signaling, epigenetic regulation, exosomal communication, macrophage polarization, T-cell exhaustion, extracellular matrix remodeling, metabolic stress, and impaired immune-cell recruitment collectively contribute to the formation of an immunosuppressive tumor niche. These mechanisms not only promote tumor progression and metastasis but also reduce the effectiveness of immunotherapy. Understanding these pathways is essential for developing more effective immune-based and combination therapies for TNBC (**Table 2**).

**Table 2:** Major tumor microenvironment-driven immune evasion mechanisms in TNBC and therapeutic opportunities.

Immune-evasion mechanism	Main TME drivers and functional consequences	Therapeutic opportunities
Checkpoint-mediated inhibition	PD-1/PD-L1, CTLA-4, LAG-3, TIM-3, TIGIT, and CD47-SIRPalpha signaling reduce T-cell activation, promote exhaustion, impair NK-cell function, and drive resistance to single-agent ICI therapy.	Combination checkpoint blockade, CD47/macrophage-phagocytosis targeting, biomarker-guided ICI selection, and pairing ICIs with chemotherapy, radiotherapy, ADCs, or targeted therapy.
Antigen-presentation loss	Reduced MHC-I expression, B2M alteration, antigen-processing defects, clonal selection, and immune editing allow immune-invisible tumor clones to persist despite immune-cell infiltration.	Restoration of antigen presentation, epigenetic priming, tumor vaccines, TCR-T approaches, and strategies that broaden tumor-antigen recognition.
Suppressive immune-cell recruitment	Tregs, MDSCs, M2-like TAMs, dysfunctional dendritic cells, tumor-associated neutrophils, and inhibitory cytokines suppress CD8+ T-cell and NK-cell cytotoxicity, weaken priming, and support metastasis.	Treg/MDSC depletion or reprogramming, TAM repolarization, dendritic-cell activation, cytokine/chemokine blockade, and myeloid-targeted combinations.
Stromal and physical exclusion	CAFs, dense ECM, collagen deposition, abnormal vasculature, hypoxia-associated fibrosis, and poor immune trafficking keep T cells outside tumor nests and reduce drug penetration.	CAF targeting, matrix normalization, FGFR/TGF-beta pathway modulation, vascular normalization, and strategies that improve immune-cell trafficking.
Metabolic and epigenetic suppression	Hypoxia, lactate accumulation, acidic pH, nutrient competition, lipid rewiring, DNA methylation, histone modification, and non-coding RNAs suppress effector-cell function and promote adaptive immune resistance.	Metabolic inhibitors, epigenetic therapy, IDO/CD73/adenosine-axis targeting, hypoxia modulation, and integration with immune checkpoint blockade.
Exosome-mediated communication	Tumor-derived extracellular vesicles carrying miRNAs, proteins, and immunosuppressive cargo condition macrophages and other immune cells, induce PD-L1, and support metastatic niche formation.	Exosome biomarker development, blockade of vesicle release or uptake, and targeting exosomal miRNA-mediated immune suppression.

### 4.6 Antigen presentation loss and immune editing

T cells are central effectors of anti-tumor immune surveillance, and their ability to recognize and eliminate malignant cells depends largely on antigen presentation through major histocompatibility complex molecules. After T-cell receptors recognize tumor-associated antigens presented on tumor cells, major histocompatibility complex class I molecules present antigenic peptides to cytotoxic T lymphocytes, leading to the activation of CD8+ T cells and the destruction of tumor cells. However, TNBC cells can evade cytotoxic immune recognition by altering or inactivating genes involved in the MHC class I antigen-presentation pathway. This loss or reduction in antigen presentation weakens cytotoxic T cells’ ability to recognize malignant cells, allowing tumor cells to persist even in an immune-infiltrated microenvironment. Recent evidence suggests that the transcription factor SOX4 is regulated by integrin αVβ6 receptors on the surface of tumor cells and contributes to resistance against T-cell-mediated cytotoxicity. Inhibition of SOX4 through integrin αVβ6 monoclonal antibodies can suppress the emergence of tumor cells with low MHC expression, increase the sensitivity of TNBC cells to cytotoxic T cells, and produce significant survival benefits in highly metastatic TNBC mouse models that respond poorly to PD-1 blockade (94). These findings indicate that immune evasion in TNBC is not limited to immune checkpoint expression but also involves defects in antigen presentation and tumor-cell-intrinsic resistance to cytotoxic immune attack.

The physical organization of the TME also contributes to immune escape. Cancer-associated fibroblasts are often distributed around tumor nests and can form a stromal barrier that prevents T cells from directly reaching and attacking tumor cells. This spatial exclusion of cytotoxic immune cells allows TNBC cells to remain protected despite the presence of anti-tumor immune activity in the surrounding tissue. Blocking the FGFR signaling pathway may disrupt this fibroblast-mediated physical barrier and inhibit the secretion of vascular cell adhesion molecule 1, thereby promoting T-cell infiltration into TNBC lesions (95). This mechanism is important because it shows that immune resistance can be produced not only through molecular inhibition but also through structural remodeling of the tumor microenvironment. Therefore, strategies that normalize or disrupt the stromal barrier may enhance the effectiveness of immunotherapy by improving immune-cell access to tumor nests.

The immune system has a dual role in cancer. On one hand, it can recognize and eliminate tumor cells; on the other hand, under certain microenvironmental conditions, it can support tumor survival, immune tolerance, and disease progression. Regulatory T cells are among the most important immunosuppressive cell populations in the tumor microenvironment. The infiltration level of regulatory T cells in the TNBC microenvironment is significantly higher than that observed in other breast cancer subtypes (96). These cells suppress excessive immune responses by secreting selected chemokines such as MIG, but this suppressive activity also creates an opportunity for tumor immune escape (97). Regulatory T cells can inhibit cytotoxic T-cell activation, reduce effector immune function, and contribute to an immunosuppressive niche that protects tumor cells from immune destruction. In addition, regulatory T cells recognize structures and molecules associated with different tumor antigens, while tumor cells can evade recognition by modifying, downregulating, or completely losing these antigens (98). This dynamic interaction between tumor-cell antigen alteration and regulatory immune suppression is a key mechanism by which TNBC maintains immune escape. Taken together, immune evasion in TNBC involves multiple interconnected layers, including impaired antigen presentation, checkpoint-mediated inhibition, cytotoxic T-cell dysfunction, fibroblast-mediated immune exclusion, regulatory T-cell infiltration, and antigenic remodeling. These mechanisms allow TNBC cells to survive even in tumors that appear immunologically active. This complexity also explains why immune checkpoint blockade alone may be insufficient for many patients. A successful therapeutic strategy may require simultaneous restoration of antigen presentation, enhancement of cytotoxic T-cell activity, reduction of regulatory T-cell-mediated suppression, disruption of fibroblast barriers, and modulation of immune-checkpoint pathways. Therefore, the TME should be viewed as a therapeutic target rather than merely a biological background.

Although the tumor microenvironment (TME)-driven mechanisms of immune evasion in TNBC—including immune checkpoint signaling, stromal exclusion, metabolic reprogramming, and myeloid cell dysfunction—are well established at the mechanistic level, their translational and clinical relevance varies considerably across pathways. Immune checkpoint inhibition targeting PD-1/PD-L1 has the strongest level of clinical validation, with demonstrated survival benefits in selected TNBC populations, establishing it as a clinically actionable axis of immune escape. In contrast, CAF-mediated immune exclusion, hypoxia-driven reprogramming, exosome-mediated intercellular communication, and metabolic competition are supported primarily by preclinical and spatial–translational studies, with limited direct clinical validation in prospective TNBC trials. Similarly, while tumor-associated macrophage (TAM) polarization and T-cell exhaustion are consistently observed in patient tumors, their causal hierarchy, temporal dominance, and predictive value for therapeutic response remain incompletely defined. Emerging mechanisms such as epigenetic regulation and non-coding RNA-mediated immune modulation provide strong biological plausibility; however, they currently lack standardized biomarkers and validated clinical stratification frameworks for routine implementation. Recent integrative analyses of the TME emphasize that spatial organization and cellular context critically determine immune functionality, further complicating direct translation of single-pathway biomarkers into clinical decision-making (99). Moreover, tumor immune phenotypes such as inflamed, excluded, and desert states are now recognized as dynamic and overlapping rather than fixed categories, highlighting the need for longitudinal and multi-modal assessment strategies (57). Collectively, these observations underscore a persistent gap between mechanistic insight and clinical translation in TNBC immuno-oncology, emphasizing the necessity for integrated multi-omics profiling and prospective biomarker-driven trials to define therapeutically relevant TME states (23).

## 5 TME-driven distant metastasis in TNBC

TNBC has a high risk of distant metastasis, which remains the principal cause of death among affected patients. Metastasis is not a random process. British surgeon Stephen Paget proposed the “seed and soil” hypothesis, suggesting that metastatic spread depends on the compatibility between tumor cells, described as “seeds”, and specific organ microenvironments, described as “soil”. According to this concept, tumor cells do not colonize distant organs by chance; rather, successful metastasis requires a supportive microenvironment that enables them to survive, adapt, proliferate, and establish secondary lesions. In TNBC, the role of the TME in metastasis has received increasing attention, particularly because interactions between cancer cells and stromal cells strongly influence invasion, dissemination, and distant colonization (58). Before tumor cells enter adjacent normal tissue and later spread to distant organs, they must first interact with tumor-associated stroma within the primary tumor. This process requires tumor cells to break through the basement membrane and enter the stromal compartment. Once inside the stroma, cancer cells encounter multiple microenvironmental components, including fibroblasts, adipocytes, mesenchymal stem cells, macrophages, and other immune cells (100). These stromal and immune cells can further enhance tumor-cell invasion and metastatic capacity through cytokine secretion, matrix remodeling, angiogenesis, inflammatory signaling, and immunosuppression (**Figure 4**).

**Figure 4:**
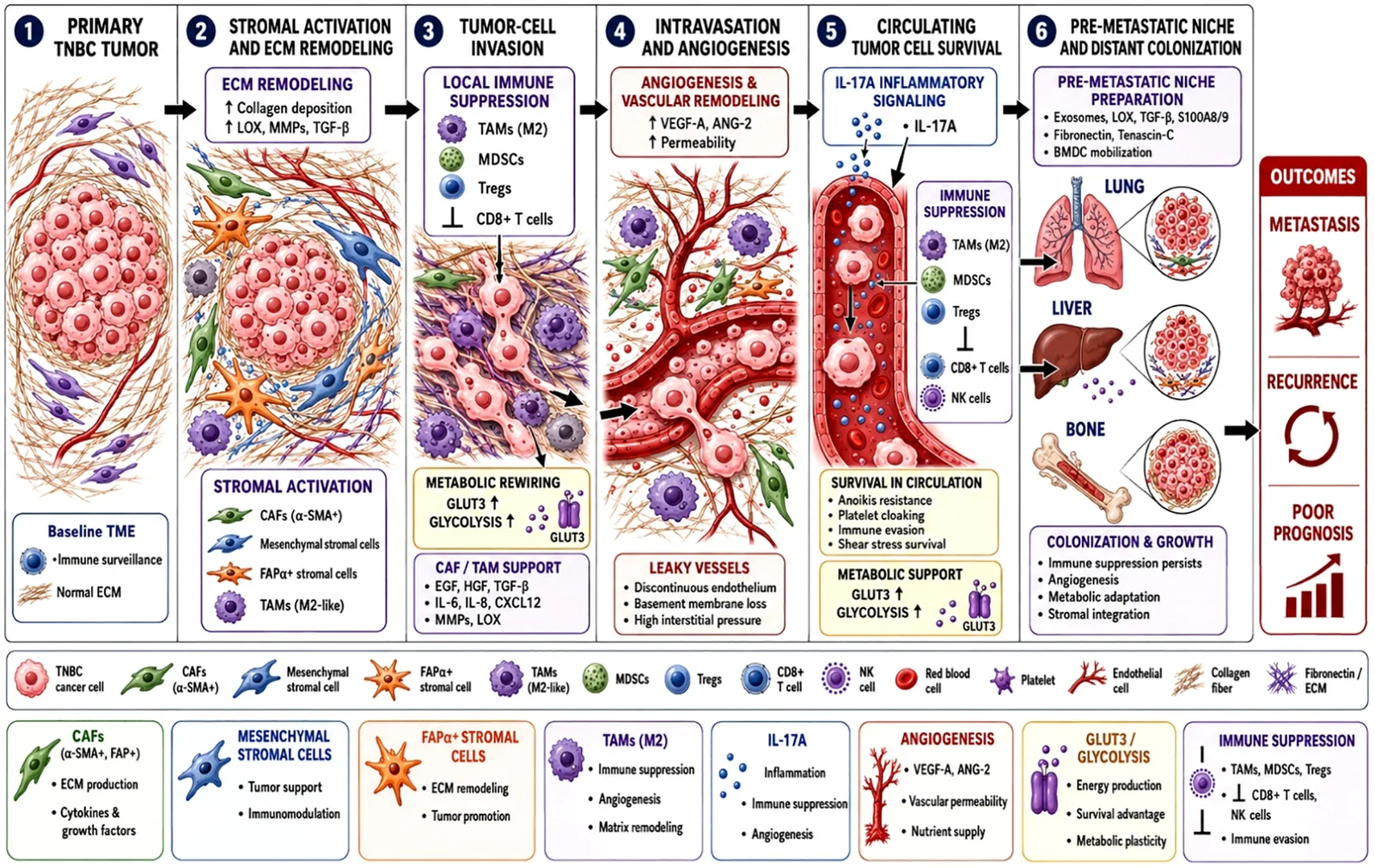
Tumor microenvironment–driven metastasis and immune-resistant progression in triple-negative breast cancer. Stepwise illustration of TNBC metastatic progression from primary tumor formation to distant organ colonization. The image (1-6) depicts sequential events, including stromal activation, extracellular matrix remodeling, tumor-cell invasion, intravasation into blood vessels, survival in circulation, formation of pre-metastatic niches, and metastatic colonization. It also highlights the roles of CAFs, tumor-associated macrophages, cytokines, angiogenic signaling, metabolic reprogramming, and immune suppression in promoting dissemination and establishing immune-resistant metastatic lesions.

Cancer-associated fibroblasts play a particularly important role in TNBC metastasis. The transformation of normal stromal fibroblasts into cancer-associated fibroblasts is a key step in the formation of a tumor-promoting microenvironment. These activated fibroblasts can directly enhance tumor-cell motility by secreting cytokines, chemokines, and growth factors into the TME. For example, cancer-associated fibroblasts can secrete CXCL12, which regulates the cytoskeleton of breast tumor cells and promotes metastatic behavior (101). Through these mechanisms, fibroblasts do not merely provide structural support; they actively participate in tumor invasion and metastatic spread. In addition, immune checkpoint molecules may contribute to metastasis through their effects on the TME. B7-H3 is expressed in tumor cells, tumor vascular endothelial cells, and professional antigen-presenting cells (102). It is specifically enriched in tumor-associated macrophages and can significantly promote metastasis by supporting extracellular matrix reconstruction and tumor angiogenesis (103). These findings suggest that immune-regulatory molecules in TNBC may have broader roles beyond immune suppression, including direct involvement in metastatic progression.

Tumor-associated mesenchymal stromal cells also participate in TNBC metastasis. These cells can arise from bone marrow mesenchymal stem cells through interactions with tumor-associated macrophages and may promote TNBC metastasis (104). Although the precise mechanisms remain unclear, current evidence indicates that tumor-associated mesenchymal stromal cells can reshape the immune and stromal environment in ways that favor metastatic progression. A key molecule involved in this process is activated fibroblast activation protein α, which is overexpressed in tumor-associated mesenchymal stromal cells. This transmembrane protein can promote the secretion of chemokine ligands by tumor-associated mesenchymal stromal cells, leading to the recruitment of tumor-associated macrophages and further polarization of these macrophages toward the M2 phenotype. M2-like macrophages are strongly associated with tumor growth, immune suppression, angiogenesis, and metastasis. Therefore, inducing apoptosis in fibroblast activation protein α-positive stromal cells may represent a promising therapeutic strategy to inhibit distant metastasis in TNBC (105). This approach is especially relevant because targeting stromal–immune interactions may more effectively suppress metastatic spread than focusing solely on tumor cells.

Inflammatory cytokines and metabolic reprogramming also contribute to TNBC metastasis. Activated T cells can release the pro-inflammatory cytokine interleukin-17A, which supports the survival of circulating tumor cells in the absence of mitosis (106). Circulating tumor cells are directly linked to metastatic dissemination and cancer recurrence, and their survival in the bloodstream is a critical step in the metastatic cascade. Interleukin-17A can also create a microenvironment that favors TNBC metastasis by stimulating endothelial fatty acid β-oxidation and promoting tumor angiogenesis (107). Increased angiogenesis provides nutrients and oxygen to rapidly growing tumors, supports metastatic colonization, and reduces ischemic necrosis during aggressive tumor progression. At the same time, TNBC often exhibits high glycolytic activity (108). Increased glucose uptake provides energy for tumor proliferation, invasion, and metastatic spread (109). Glucose transporter protein 3 is particularly elevated in TNBC and can induce regulatory factors within the TME that promote an inflammatory microenvironment, activate macrophages, and support distant metastasis of tumor cells (110). These findings demonstrate that metabolic alterations are closely connected to immune regulation and metastatic behavior in TNBC.

## 6 Clinical landscape of immunotherapy and TME-directed therapeutic strategies

Immune checkpoint inhibitors have changed the therapeutic landscape of TNBC, particularly when combined with chemotherapy. Clinical evidence indicates that PD-1/PD-L1 blockade can improve outcomes in selected patients, especially those with PD-L1-positive tumors or immune-inflamed microenvironments. In the IMpassion130 trial, atezolizumab plus nab-paclitaxel prolonged progression-free survival in patients with advanced or metastatic TNBC, with exploratory survival benefit mainly observed in the PD-L1-positive subgroup. The final overall survival analysis further supported the clinical relevance of PD-L1-positive immune-enriched tumors, although the intention-to-treat population did not show a statistically significant overall survival advantage. In KEYNOTE-355, pembrolizumab plus chemotherapy significantly improved progression-free survival and overall survival in patients with previously untreated, locally recurrent, inoperable, or metastatic TNBC whose tumors expressed PD-L1. In early-stage high-risk TNBC, KEYNOTE-522 showed that pembrolizumab combined with neoadjuvant chemotherapy, followed by adjuvant pembrolizumab, improved pathological complete response, event-free survival, and later overall survival, as shown in **Table 3**.

**Table 3:** Key clinical trials evaluating immunotherapy in triple-negative breast cancer (TNBC).

Clinical trial	Therapy	Setting	Main outcome
IMpassion130	Atezolizumab + nab-paclitaxel	Metastatic TNBC	Improved PFS in PD-L1+ subgroup; OS benefit not significant in overall population
IMpassion131	Atezolizumab + paclitaxel	Metastatic TNBC	No significant improvement in PFS or OS
KEYNOTE-355	Pembrolizumab + chemotherapy	Metastatic TNBC	Significant improvement in PFS and OS in PD-L1+ patients
KEYNOTE-522	Pembrolizumab + neoadjuvant chemotherapy	Early-stage TNBC	Increased pCR rate and improved event-free survival
IMpassion031	Atezolizumab + neoadjuvant chemotherapy	Early-stage TNBC	Increased pathological complete response rate
TONIC trial	Nivolumab ± induction therapy	Metastatic TNBC	Induction therapy increased response to PD-1 blockade
GeparNuevo	Durvalumab + chemotherapy	Early-stage TNBC	Trend toward improved pCR and long-term survival benefit

Clinical trial evidence collectively demonstrates that immune checkpoint inhibitors provide meaningful clinical benefit in selected patients with TNBC, particularly those with PD-L1-positive tumors or immune-inflamed tumor microenvironments. However, the magnitude of benefit varies significantly across trials, reflecting heterogeneity in trial design, chemotherapy backbone, biomarker selection, and tumor immune contexture. While IMpassion130 and KEYNOTE-355 established the clinical utility of immunotherapy in metastatic TNBC, IMpassion131 highlighted the dependency of therapeutic efficacy on chemotherapy partners. In early-stage disease, trials such as KEYNOTE-522 and IMpassion031 demonstrate that combining immune checkpoint blockade with neoadjuvant chemotherapy can enhance pathological response rates and improve long-term outcomes. Collectively, these findings underscore that immunotherapy efficacy in TNBC is strongly influenced by tumor microenvironmental factors, reinforcing the need for biomarker-guided patient selection and rational combination strategies.

Accurate stratification of triple-negative breast cancer (TNBC) patients based on tumor microenvironment (TME) complexity requires integrating advanced spatial, single-cell, and multi-omics technologies to capture both cellular diversity and functional states. Single-cell RNA sequencing enables high-resolution dissection of tumor, stromal, and immune compartments, revealing distinct cellular states associated with immune activation and suppression. Spatial transcriptomics preserves tissue architecture and allows mapping of immune-cell localization, stromal interactions, and immune-exclusion niches within TNBC lesions. Recent spatial transcriptomics studies have demonstrated that cancer-associated fibroblast niches can orchestrate dual stromal and immune barriers, reinforcing immune exclusion in solid tumors (111). Imaging mass cytometry and multiplex immunohistochemistry further provide highly multiplexed protein-level profiling with spatial resolution, enabling quantitative assessment of immune infiltration, checkpoint expression, and spatial immune architecture. Emerging spatial multi-omics frameworks integrating transcriptomic, proteomic, and metabolomic layers have demonstrated improved capability in defining functional tumor immune ecosystems and identifying clinically relevant immune phenotypes (112, 113). In addition, liquid biopsy approaches, including circulating tumor DNA, circulating tumor cells, and exosomal profiling, enable non-invasive longitudinal monitoring of tumor evolution and immune escape dynamics (114, 115). Finally, artificial intelligence- and machine learning-based integrative models are increasingly being developed to combine multimodal datasets to robustly predict TME states and immunotherapy response, thereby enabling precise stratification of TNBC into immune-inflamed, immune-excluded, and immune-desert phenotypes. Fusion-network-based computational models have also been developed to identify prognostic and heterogeneous biomarkers in breast cancer, supporting improved patient stratification strategies (116).

Chemo-immunotherapy is particularly relevant in TNBC because chemotherapy can enhance immune responsiveness through mechanisms beyond direct cytotoxicity. Chemotherapy may increase tumor antigen release, promote immunogenic cell death, improve dendritic cell priming, reduce selected suppressive immune populations, and increase the vulnerability of tumor cells to immune-mediated clearance. However, the same treatment pressure can also select resistant clones, induce lymphodepletion, promote compensatory checkpoint expression, and reshape the TME toward immune suppression. Therefore, the timing, sequence, and selection of chemotherapy partners are critical for maximizing immunotherapy benefit. Although endocrine-immune combinations are more directly applicable to hormone receptor-positive breast cancer, they remain important for the broader breast cancer immune-evasion field because they show how subtype-specific tumor biology influences immunotherapy design. In TNBC, where endocrine therapy is not applicable, the corresponding challenge is to identify rational immune partners, including chemotherapy, antibody–drug conjugates, PARP inhibitors, radiotherapy, metabolic inhibitors, macrophage-targeting agents, and stromal-modulating therapies (117–119).

Antibody-based therapies are also expanding the therapeutic options for TNBC. Monoclonal antibodies can target immune checkpoints, stromal mediators, cytokine and chemokine pathways, or tumor-associated antigens, thereby modulating both tumor-intrinsic and TME-driven resistance mechanisms. Antibody–drug conjugates represent a particularly important advance because they combine antibody specificity with cytotoxic payload delivery. Recent nanoformulation strategies using disulfide bond–driven prodrug assemblies have further improved drug stability and tumor-targeted delivery in breast cancer therapy (120). Sacituzumab govitecan, which targets Trop-2 and delivers the SN-38 payload, has demonstrated significant clinical benefit in metastatic TNBC compared with single-agent chemotherapy and is now one of the most important ADC-based options for heavily pretreated disease. Beyond direct tumor-cell killing, ADCs may also contribute to TME remodeling through immunogenic cell death, antigen release, immune-cell recruitment, and bystander killing of antigen-heterogeneous tumor cells. Emerging antibody formats, including bispecific and multispecific antibodies, may further overcome immune resistance by redirecting T cells or NK cells toward tumor cells independently of conventional antigen presentation. These approaches are highly relevant for TNBC because antigen heterogeneity, immune-cell exclusion, and defective MHC-I presentation can limit the effectiveness of conventional immune checkpoint blockade (121).

mRNA-based immunotherapies represent another emerging strategy for reprogramming the TNBC immune microenvironment. These platforms are attractive because they are programmable, transient, non-integrating, and adaptable to patient-specific tumor antigens. In TNBC, mRNA strategies are being developed in three major directions: personalized vaccines encoding tumor-associated antigens or neoantigens; mRNA-engineered immune cells, such as CAR-T or TCR-T cells; and mRNA-encoded immunomodulators that deliver cytokines or costimulatory ligands to reshape the TME. Candidate antigens and targets include MAGE-A3, NY-ESO-1, TP53- or BRCA-related neoantigens, ROR1, Trop-2, Claudin 6, and Nectin-4, while immunomodulatory payloads include IL-12, GM-CSF, OX40L, and 4-1BBL. Lipid nanoparticles and tumor-penetrating delivery systems can protect mRNA cargo, improve cellular uptake, and support tumor- or lymphoid-targeted delivery. By promoting dendritic cell maturation, CD8+ T-cell activation, macrophage reprogramming, and the conversion of immune-cold tumors into immune-inflamed tumors, mRNA therapeutics may help overcome immune exclusion and antigenic heterogeneity in TNBC (73). Advanced immunotherapy strategies are also being explored to overcome immune evasion mechanisms that limit the efficacy of conventional checkpoint blockade. Tumor vaccines aim to increase immune recognition by presenting tumor-associated antigens or patient-specific neoantigens to activate durable T-cell responses. CAR-T and TCR-T therapies offer another strategy by engineering immune cells to recognize tumor-associated targets and directly eliminate malignant cells. However, their success in TNBC depends on overcoming antigen heterogeneity, limited trafficking into solid tumors, stromal exclusion, T-cell exhaustion, suppressive cytokines, and on-target/off-tumor toxicity. Bispecific antibodies and immune-cell engagers may help address some of these barriers by redirecting T cells or natural killer cells against tumor cells without fully relying on endogenous antigen presentation. These approaches are especially relevant for immune-cold or immune-excluded TNBC, where the major therapeutic goal is not only to activate immune cells but also to guide them into the tumor, maintain their function, and prevent their inactivation by a suppressive TME (122–126).

Radiotherapy also has important immunological implications in TNBC because it can remodel the TME in both immune-stimulatory and immunosuppressive directions. On the one hand, radiotherapy can induce DNA damage, immunogenic cell death, ATP and HMGB1 release, calreticulin exposure, damage-associated molecular pattern signaling, cGAS-STING pathway activation, dendritic-cell maturation, antigen presentation, and CD8+ T-cell and NK-cell activation. These effects can increase tumor immunogenicity and provide a rationale for combining radiotherapy with immune checkpoint inhibitors. On the other hand, radiotherapy can also induce immunosuppressive cytokines such as TGF-β and IL-10, promote lymphopenia, recruit myeloid-derived suppressor cells, expand regulatory T cells, and shift macrophages toward M2-like phenotypes, thereby contributing to immune escape, recurrence, and radioresistance. Current strategies that combine radiotherapy with anti-PD-1/PD-L1 therapy, anti-CTLA-4 therapy, PARP inhibitors, PI3K inhibitors, MDSC blockade, or macrophage-targeting approaches aim to preserve the immune-stimulating effects of radiotherapy while limiting radiation-induced immunosuppression (127).

Biomarker-guided patient selection is essential for improving the precision of TNBC immunotherapy. PD-L1 expression and stromal tumor-infiltrating lymphocytes remain among the most clinically relevant markers. Still, their predictive value is limited by assay variability, spatial heterogeneity, temporal changes during treatment, and differences between primary and metastatic lesions. Additional biomarkers under investigation include tumor mutational burden, homologous recombination deficiency, BRCA1/2 status, immune gene signatures, MHC-I expression, antigen-presentation machinery, T-cell exhaustion markers, myeloid-cell infiltration, CD163+/CD68+ macrophage density, regulatory T-cell infiltration, MDSC abundance, FASN, LDHA, IDO1, CD73, and non-coding RNA signatures. Single-cell RNA sequencing, spatial transcriptomics, multiplex imaging, proteomics, and artificial intelligence-supported multi-omics integration may enable more accurate classification of immune-responsive and immune-resistant TNBC states (**Figure 5**). Deep learning-based imaging approaches have also demonstrated high accuracy in breast lesion detection using ultrasound image analysis, supporting AI-driven diagnostic stratification in clinical practice. Adaptive fusion neural networks have also been applied to high-resolution medical image reconstruction, improving diagnostic imaging quality in oncological applications (128). AI-based integrative prognostic models have also been successfully applied in other malignancies such as melanoma to identify stress-response–associated gene signatures (129). These tools can support adaptive clinical trial design and individualized therapy selection based on the immune, stromal, metabolic, and spatial organization of each tumor (130, 131).

**Figure 5:**
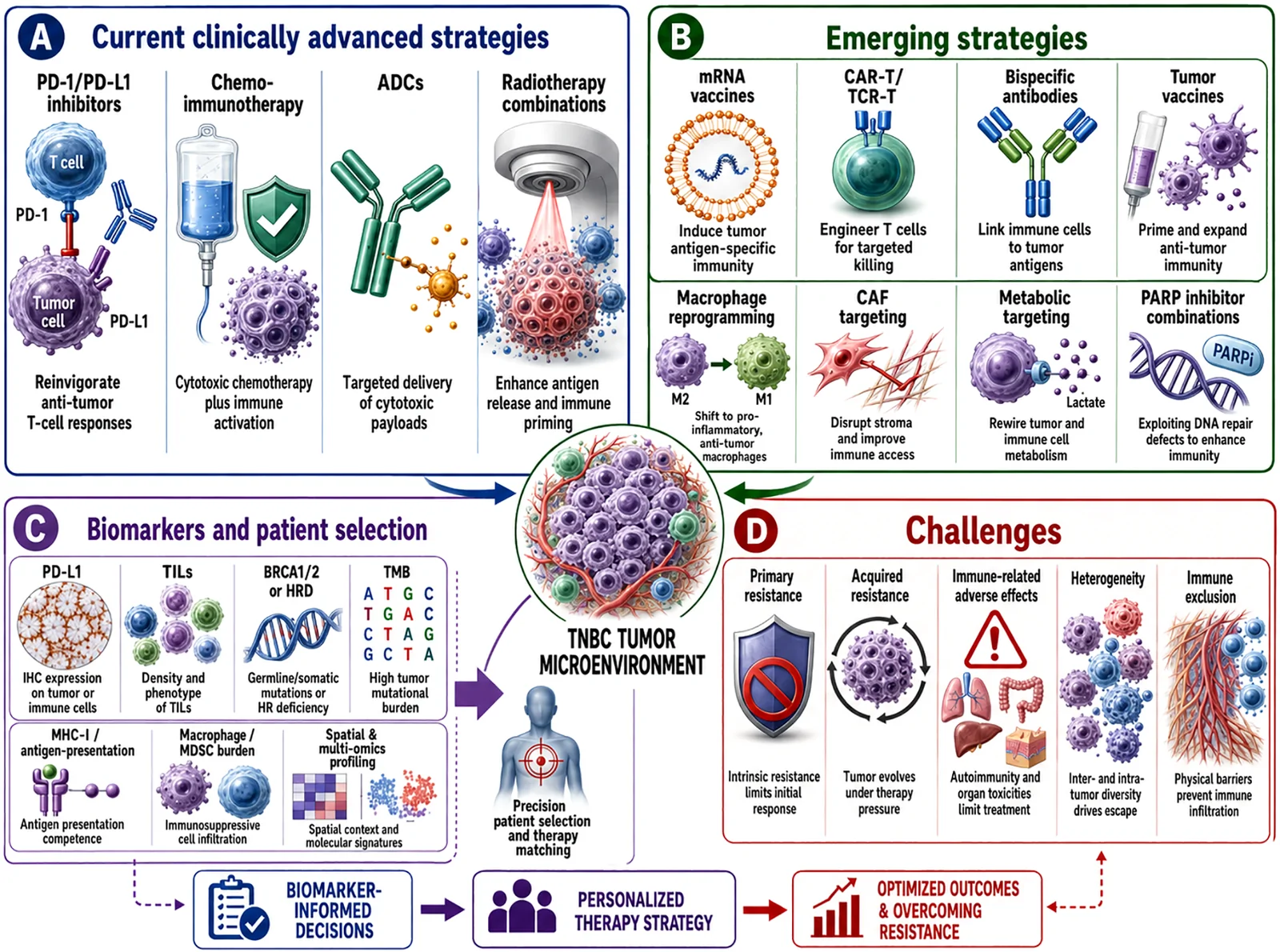
Clinical immunotherapy landscape and tumor microenvironment–directed therapeutic strategies in triple-negative breast cancer. Schematic overview of current and emerging therapeutic approaches targeting TNBC, integrating immune checkpoint blockade, combination chemotherapy–immunotherapy, stromal reprogramming, metabolic targeting, and myeloid-cell modulation strategies. The panels **(A–D)** also summarizes biomarker-driven patient stratification approaches, including PD-L1 expression, tumor-infiltrating lymphocytes, genomic and transcriptomic signatures, and multi-omics profiling. In addition, it highlights the role of advanced technologies such as single-cell sequencing, spatial transcriptomics, multiplex imaging, and artificial intelligence–based models in guiding precision immunotherapy and improving clinical outcomes.

### 6.1 Single-cell transcriptomics, AI-driven biomarker discovery, and emerging immunotherapies in TNBC

Single-cell RNA sequencing (scRNA-seq) has become a transformative technology for dissecting tumor and immune cell heterogeneity in triple-negative breast cancer (TNBC), enabling high-resolution identification of rare immune populations, exhausted T-cell states, and dynamic tumor–immune interactions that are not observable in bulk transcriptomic analyses. Integration of scRNA-seq with spatial transcriptomics further enhances the ability to map immune-cell localization, stromal architecture, and immune-exclusion niches while preserving spatial context within the tumor microenvironment. These approaches provide a more comprehensive understanding of functional tumor ecosystems and immune evasion strategies at single-cell resolution (132). In parallel, artificial intelligence (AI) and machine learning-based frameworks are increasingly being applied to multi-omics datasets, including genomic, transcriptomic, imaging, and clinical data, to identify predictive biomarkers and generate integrative models to predict immunotherapy response in TNBC. Such computational approaches allow stratification of patients based on complex tumor immune ecosystem signatures rather than single-gene biomarkers, improving predictive accuracy for immunotherapy outcomes (133). In addition, emerging immunotherapeutic strategies are expanding the TNBC treatment landscape. Bispecific antibodies and T-cell-engaging constructs are designed to simultaneously bind tumor antigens and immune effector cells, thereby enhancing cytotoxic immune responses and overcoming immune suppression within the tumor microenvironment (126). Nanoparticle-based sono-immunotherapy platforms have recently been developed to enhance immune activation and therapeutic efficacy in pancreatic cancer models, suggesting broader applicability of physical–immune combination strategies (134, 135). Furthermore, personalized neoantigen vaccines represent a promising precision immunotherapy strategy that aims to elicit robust, durable T-cell responses against patient-specific tumor mutations, thereby enhancing immunogenicity in otherwise immune-cold TNBC subtypes. Collectively, these advances highlight a paradigm shift toward precision immuno-oncology, integrating high-resolution cellular profiling with next-generation immunotherapies to improve patient stratification and therapeutic efficacy in TNBC.

## 7 Future perspectives and therapeutic opportunities

Future therapeutic development in TNBC should move from single-target immune checkpoint inhibition toward integrated TME-directed strategies. The central challenge is that overlapping mechanisms, including antigen-presentation loss, PD-L1 and alternative checkpoint activation, T-cell exhaustion, MDSC and Treg expansion, M2 macrophage polarization, CAF-mediated immune exclusion, metabolic competition, epigenetic silencing, clonal selection, and treatment-induced immune adaptation, maintain TNBC immune evasion. Therefore, future treatment design should combine immune checkpoint blockade with rational partners, including ADCs, mRNA vaccines, PARP inhibitors, radiotherapy, macrophage-reprogramming agents, CAF-targeting agents, metabolic inhibitors, cytokine/chemokine blockade, tumor vaccines, bispecific antibodies, and adoptive immune-cell therapies. This approach is more consistent with the biological complexity of TNBC and may improve the conversion of immune-cold or immune-excluded tumors into immune-responsive tumors.

TNBC is widely considered one of the most aggressive subtypes of breast cancer. Because it lacks ER, PR, and HER2 expression, specific therapeutic targets are limited, and chemotherapy has traditionally remained the major systemic treatment option. However, chemotherapy alone is often insufficient because TNBC is prone to drug resistance, recurrence, and metastasis. The close relationship between TNBC cells and the TME provides an important explanation for these clinical challenges. TNBC and its microenvironment are interdependent, mutually reinforcing, and dynamically competitive. Tumor cells reshape the TME to support survival and immune escape, while the TME provides signals that promote tumor progression, metastasis, and treatment resistance. Therefore, research on the TME has become a central direction in current tumor biology and translational cancer research.

In recent years, advances in tumor cytology, molecular biology, immunology, and multi-omics technologies have deepened the understanding of interactions between TNBC and the TME. These advances have clarified the significance of immune cells, stromal cells, extracellular matrix remodeling, cytokine networks, immune-checkpoint pathways, metabolic reprogramming, and exosomal communication in TNBC occurrence, progression, metastasis, prevention, treatment, and prognosis. The clinical development of immune checkpoint inhibitors has also increased attention to the TNBC immune microenvironment. The approval of PD-1/PD-L1-targeted immunotherapy for selected TNBC patients has brought new hope and has confirmed the clinical relevance of TME-focused research. These developments suggest that the immune microenvironment is not only a biological feature of TNBC but also a practical therapeutic target.

Despite this progress, many mechanisms underlying TME-mediated immune evasion, metastasis, and treatment resistance remain incompletely understood. Not all patients benefit from immunotherapy, and many tumors develop primary or acquired resistance. This indicates that immune checkpoint blockade alone cannot fully overcome the complexity of the TNBC microenvironment. Future research should focus on identifying reliable biomarkers for patient selection, clarifying the mechanisms that distinguish immune-responsive from immune-resistant tumors, and developing combination strategies that target multiple components of the TME. Potential approaches include combining immune checkpoint inhibitors with chemotherapy, antibody-drug conjugates, targeted therapy, anti-angiogenic therapy, metabolic inhibitors, macrophage-modulating agents, cancer-associated fibroblast-targeting strategies, tumor vaccines, adoptive T-cell therapies, or agents that restore antigen presentation and T-cell infiltration.

Overall, the TME is deeply involved in the biological aggressiveness, immune escape, metastatic behavior, and treatment resistance of TNBC. Continued investigation of TME-related mechanisms is expected to identify new therapeutic targets and improve treatment options for patients. Although major challenges remain, the rapid development of immunotherapy and microenvironment-directed strategies provides a strong foundation for improving TNBC management. With further discovery of immune-evasion pathways, metastasis-related stromal mechanisms, and predictive biomarkers, TNBC may gradually become more controllable and treatable through individualized and combination-based therapeutic approaches. Therefore, future progress in TNBC immunotherapy will depend on moving beyond single immune checkpoint blockade toward integrated strategies that simultaneously restore immune recognition, reverse suppressive TME signaling, improve immune cell infiltration, control therapy-induced resistance, manage immune-related toxicity, and personalize treatment based on spatial, molecular, metabolic, and immunological biomarkers.
